# Adaptation of mammalian host-pathogen interactions in a changing arctic environment

**DOI:** 10.1186/1751-0147-53-17

**Published:** 2011-03-11

**Authors:** Karsten Hueffer, Todd M O'Hara, Erich H Follmann

**Affiliations:** 1 Molecular Ecology of Infectious Agents Laboratory University of Alaska Fairbanks, 902 N. Koyukuk Dr., Fairbanks AK 99775, USA; 2 Institute of Arctic Biology, Department of Biology and Wildlife, University of Alaska Fairbanks, 902 N. Koyukuk Dr., Fairbanks AK 99775, USA

## Abstract

Many arctic mammals are adapted to live year-round in extreme environments with low winter temperatures and great seasonal variations in key variables (e.g. sunlight, food, temperature, moisture). The interaction between hosts and pathogens in high northern latitudes is not very well understood with respect to intra-annual cycles (seasons). The annual cycles of interacting pathogen and host biology is regulated in part by highly synchronized temperature and photoperiod changes during seasonal transitions (e.g., freezeup and breakup). With a warming climate, only one of these key biological cues will undergo drastic changes, while the other will remain fixed. This uncoupling can theoretically have drastic consequences on host-pathogen interactions. These poorly understood cues together with a changing climate by itself will challenge host populations that are adapted to pathogens under the historic and current climate regime. We will review adaptations of both host and pathogens to the extreme conditions at high latitudes and explore some potential consequences of rapid changes in the Arctic.

## Introduction

**Figure 1:**
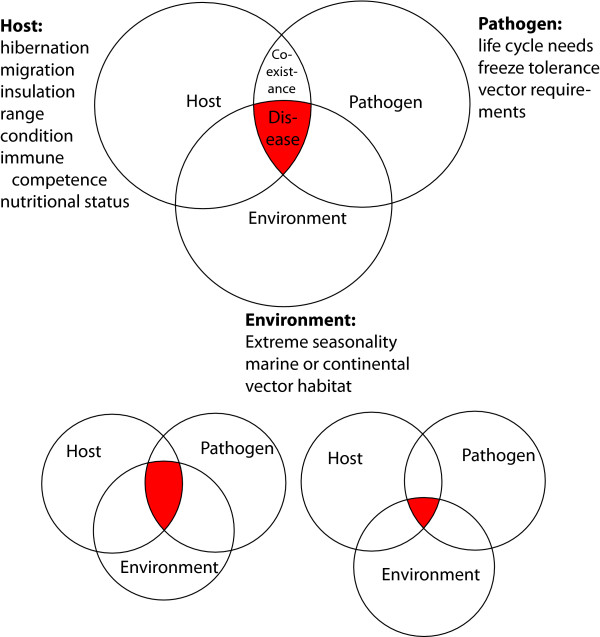
**The epidemiological triad: Interaction of host, environment, and pathogen determine disease and pathogen ecology**. Some factors influencing the components of the triad are shown for the current epidemiological triad (top panel). Potential changes in the epidemiological triad are shown in the bottom panels through a changing climate and a resulting change in the environmental component of the triad.

We review the current knowledge on arctic mammalian hosts, pathogens, and "climate change" from the epidemiologic triad perspective (Host - Pathogen - Environment). These complex interactions require better understanding to develop conceptual models and possible predictive mechanisms that impact wildlife and public health (zoonoses) management for a changing arctic environment. Temperature and photoperiod changes are major cues regulating extreme biological changes during the annual cycle for many arctic resident mammals, most notably at seasonal transitions during spring and autumn. Uncoupling of major cues in seasonal rhythms due to changing climate could have drastic consequences for behavior of arctic animals and pathogens, thus altering the interactions of the components of the epidemiologic triad (Figure 1). We explore general adaptation to high latitudes in host-pathogen interactions and possible consequences of a decoupling of seasonal changes (e.g. warmer temperatures during key time periods).

Extreme conditions characterize the arctic environment. Winter temperatures regularly reach -50°C and lower, dominant snow cover, and photoperiod changes (0 hours of direct sunlight in winter and 24 hours in summer) demand specialized adaptations. Some of the physiological and behavioral adaptations to this extreme environment are only poorly understood especially in a predictive context of a changing Arctic. In the context of a warming Arctic the stressors related to heat during summer months is even less well understood for these species adapted to high latitude conditions, as most studies have focused on cold adaptation rather than adaptation to extreme seasonal changes.

Climate change has, and is predicted to continue to have, the greatest magnitude at high latitudes [1]. A recent study reconstructed the arctic climate for the last 2000 years and the decade from 1990 to 2000 was the warmest with 4 out of 5 of the warmest decades during the period of 1950 to 2000 [2]. A recent Intergovernmental Panel on Climate Change (IPCC) report [3] predicts mean temperature rise in the Arctic for the 21^st ^century will be 5°C (range 2.8°C to 7.8°C), the largest changes to occur during winter. An increase in precipitation, especially during the winter months, is also predicted. Drastic consequences for mammals living at high northern latitudes will result from a decrease in sea ice, thawing permafrost, change in vegetation and earlier spring melt ("breakup") and later "freeze up" with a fixed annual photoperiod cycle.

As a result of these climatic changes an increase of human development and activity (resource exploration and shipping) in the Arctic is expected. The prospect of an ice-free Northwest Passage will certainly increase maritime traffic and efforts to extract resources such as minerals, oil and natural gas in the high Arctic. This human development will increase contact of wildlife with humans and their domesticated animals, including their biological wastes. Such an increased interaction will greatly affect arctic fauna [4,5].

Anticipated changes in the Arctic have generated concern for wildlife population health. As an example, in 2006 the polar bear (*Ursus maritimus*) was characterized as a vulnerable species by the International Union for Conservation of Nature and Natural Resources (IUCN), mainly due to concerns regarding climate change. In 2008 the polar bear was listed as threatened in the USA according to the Endangered Species Act, because of observed and anticipated further decrease of sea ice extent [6].

Most recent discussions focus on habitat loss and changes in food availability, however, infectious disease agents are also predicted to change their impact on mammalian populations. Predicted changes include the expansion of arthropod vectors into northern habitats as well as increased pathogen transmission through direct and indirect contact with humans and their domesticated animals. Some pathogens will decrease in prevalence and impact as well with the shifts in environmental conditions [7].

Infectious agents and hosts have adapted to high latitude extreme conditions. These host-pathogen interactions cannot be reliably extrapolated from known lower latitude interactions of related species. Some changes in connection with warming trends have already been observed with a shortened life cycle of the nematode parasite *Umingmakstrongylus pallikuukensis *of musk ox (*Ovibos moschatus moschatus*) and a resulting increase in parasite burden [8]. In this host species an increase in bacterial lung infections has been linked to warmer temperatures as one factor in increased mortality in Norway [9]. In addition, the increased occurrence of the filarioid nematode *Setaria tundra *in Finnish reindeer (*Rangifer tarandus*) has recently been linked to higher temperatures in years of outbreaks as well as increased temperatures in the preceding year [10].

Wild mammals play a significant role in human culture and nutrition in the Arctic, as a subsistence lifestyle is still common [11]. Changes in infectious disease ecology will have significant implications for food security of underserved indigenous populations in remote arctic areas with known health disparities. The potential for introduction of zoonotic agents can also threaten general public health and food security. This is especially true because traditional knowledge does not necessarily provide the tools and behaviors to appropriately address these potential new (exotic) infectious agents threats.

We review examples of adaptations of arctic host - pathogen interactions and general features to identify major gaps in our knowledge of high latitude host-pathogen interaction.

## Environment

The Arctic is defined by some as the area of the planet North of the Arctic Circle (Latitude 66° 33' 39"). However, other definitions of the Arctic also exist, such as the 10°C July isotherm or the northern tree line. It is comprised of the Arctic Ocean, the northern part of the Pacific and Atlantic oceans with large coastal areas, and terrestrial ecosystems [12]. The vegetation is characterized by boreal forest in the southern Arctic and tundra in the northern, and coastal areas. Portions of the marine environment are ice covered, either year round or throughout the winter months with latitudinal variation based on oceanographic influences. Many areas of the Arctic Ocean and proximate marine regions are highly productive continental shelf habitats [12], thus of great interest to conservationists, commercial fisheries and subsistence users.

The climate is characterized by low mean annual air temperatures, which varies widely over the Arctic with winter temperatures in coastal areas generally higher than in areas with a continental (interior) climate; and the opposite during summer. These effects are reflected in the mean annual temperatures of Murmansk (0°C), Barrow (-12°C), Tromsø (3.5 °C), and Verkhoyansk (-15°C) for communities at a similar relative latitude [13].

**Figure 2:**
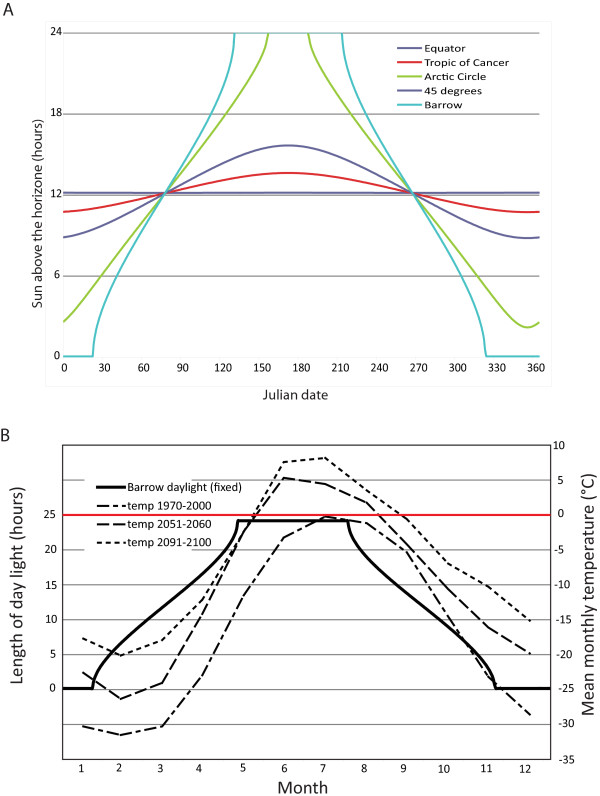
**Daylight length and temperature at high latitudes: A) Daylight length at different latitudes of the Northern hemisphere**. B) Daylight length and current as well as predicted temperatures for Barrow, Alaska (USA). Predicted mean monthly temperatures were obtained using the Echam5 model [58] for the years 2051-2060 and 2091-2100 and compared to measured temperatures in Barrow for the years 1970-2000. The red line indicates 0 degree Celsius.

Extreme seasonal variation in temperature is caused by a drastic change in photoperiod over the seasons, with 24 hours of daylight and a full day of darkness at the solstices [12] (Figure 2A). The large changes in temperature in conjunction with changes in photoperiod lead to drastic changes in ecosystem productivity at high latitudes during the annual cycle and drive major changes to mammalian behavior, abundance, movement, and distribution in the Arctic. This reduced production during winter is compounded by snow and ice cover further reducing food availability to many terrestrial mammals, especially herbivores. Winter sea ice limits entry of some mammals.

While major changes in arctic temperatures are expected, the photoperiod will stay constant (Figure 2B). The resulting uncoupling of two major cues in seasonal rhythms could have drastic consequences for the behavior of arctic animals, pathogens and associated vectors, intermediate hosts, and fomites. Detailed consequences of such an uncoupling cannot be predicted with our current knowledge of the role these cues play in regulating seasonal changes in both hosts and pathogens.

The arctic environment produces a habitat where only relatively few species can successfully compete year round. Overall arctic biodiversity is relatively low compared to more temperate and tropic climates. Lower biodiversity makes the ecosystem more susceptible as the pool of possible genetic solutions is not as diverse to adapt to changes in the environment [14,15]. Together with the predicted larger impact of anthropogenic climate change at higher latitudes this suggests the arctic and subarctic environments are less resilient to these changes. However, for some systems this argument might not hold true entirely [16].

## Host

Mammals in the Arctic are adapted to both the extreme absolute temperatures during winter as well as the drastic changes over the annual cycle. Mammals have evolved different mechanisms to survive and thrive under these extreme conditions; with true hibernation probably being the most extreme physiological adaptation and these adaptations cannot necessarily be extrapolated from knowledge about closely related species at lower latitudes.

The polar bear is an example of differences compared to other bear species at lower latitudes, especially the brown bear (*Ursus arcos*, which evolved at lower latitudes). Only pregnant female polar bears den during the winter, while non-pregnant female and male polar bears spend the winter on the sea ice and hunt seals or scavenge marine mammal carcasses [17]. In contrast, both sexes of brown and black bears hibernate except perhaps at the most southerly latitudes in their distribution where temperatures are moderate and food is not limited [17].

### Hibernation

Hibernation behavior varies between species. For many mammals, this event is highly regulated from a temporal perspective with critical factors required for a successful overwintering (survival and/or reproduction). The Arctic ground squirrel (*Spermophilus parryii*) has one of the most physiologically extreme hibernation behaviors. Generally hibernation is characterized by reduced metabolism and a reduced body temperature. During torpor, hibernating animals maintain low body temperatures for several months, interrupted only by sporadic, short (<24 hours) spontaneous euthermic arousals at intervals of several weeks. The arctic ground squirrel reduces its core body temperatures to below 0°C [18]. The extremely low body temperatures of the arctic ground squirrel are still actively controlled by the animal, as their body temperature is still above the hibernaculum's ambient temperature and they respond to core body temperatures below -4°C by warming.

During low temperature of torpor, immune functions are suppressed [19]. Therefore, hibernating animals provide a unique host environment that is different from euthermic animals that are normally studied when assessing susceptibility to pathogens. In addition, knowledge of lowered immune function in hibernating animals is based mainly on findings in the 13 lined ground squirrel (*Spermophilus tridecemlineatus)*, the golden-mantled ground squirrel (S*permophilus lateralis)*, and other hibernators that hibernate at relatively higher core body temperatures than *S. parryii*. The suppressed immune function was illustrated in two infection studies using *S. lateralis *and *S. tridecemlineatus*. In these studies, infections with Coxsackie B-3 [20] virus and *Mycobacterium leprae *[21] were highly pathogenic in hibernating ground squirrels but not in euthermic active animals. The number of circulating white blood cells [22,23] and the ability to produce antibodies is reduced during torpor and therefore adaptive immune functions are presumably also reduced [24,25]. In addition, the complement system activity varied greatly in the golden-mantled ground squirrel among stages of the annual cycle [26]. The activity was lowest during torpor and highest in summer active animals followed by animals in interbout arousal. In contrast to systemic immune functions intestinal immune functions such as intra epithelial leukocyte numbers in the intestinal wall increased during torpor indicating shift to maintain the barrier function of the gastrointestinal tract during hibernation periods [27].

While low body temperature reduces the replication of many pathogenic microbes, some pathogens such as *Listeria monocytogenes *and *Campylobacter jejuni *replicate at these low temperatures. In addition, inter-torpor euthermic arousals would allow for significant pathogen replication. However, during these arousals the immune responsiveness of the hibernating animals also is elevated. As already mentioned, one has to be cautious in extrapolating findings from non-arctic species to species adapted to the extreme high latitude conditions. Warmer temperatures and the associated uncoupling of temperature cues and photoperiod cues could potentially expose hibernators to disease agents during periods of decreased immunocompetence (Figure 1B and Figure 2B). One example of severe infection during hibernation is the white nose syndrome in bats at lower latitudes, a fungal infection that has led to the collapse of many bat colonies during winter [28].

While hibernation is controlled by an internal annual rhythm [29], this induction of physiological changes can be overcome by some environmental factors. Changes in hibernation behavior have been reported for male grizzly bears in Yellowstone National Park, which entered dens later in recent years [30]. With a predicted increase in snowfall, an earlier arousal into a warmer and wetter environment can have severe consequences for herbivores emerging from hibernation into a habitat where forage is not accessible due to deeper snow cover. This phenomenon has already been observed at high altitudes [31] and animals were able to adjust by moving down slope into areas with less snow. At high latitudes, this strategy would not be feasible, as areas with less snow cover would be located at much longer distances from the denning sites compared to high altitude where areas with low snow cover are available in relatively close proximity (e.g. down slope, southerly aspect)

### Immune gene diversity

Immune response genes, especially those involved in pathogen presentation, are highly polymorphic. This polymorphism is driven by the need to interact with a plethora of pathogenic organisms and mount appropriate defensive responses. For the Arctic with a low biodiversity Geist (1985) proposed that exposure to a reduced pathogen spectrum leads to decreased ability to resist a wide range of pathogens [32]. Several studies indicate low levels of polymorphism at mitochondrial and MHC loci [33-36]. In fact monomorphism was found at three loci in musk ox [37]. However, this hypothesis does not hold true for polar bears [17]. Apart from the lower parasite pressure, genetic bottlenecks could also influence diversity in immune genes.

An additional factor complicating rapid evolutionary adaptation in arctic mammals, especially large mammals, is the long life span and later reproductive maturity. Some arctic whale species reach up to 200 years in age and do not become reproductively active until 20 years of age or older [38]. In conjunction with low diversity and relative isolation for long periods of their evolutionary history, this long generation time can, for some species, greatly reduce their ability to respond to new pathogens and a changing environment in general.

## Pathogen

As expected some pathogens in the high Arctic are similarly adapted to the extreme environment as their hosts. This is especially true for pathogens with a life stage in an invertebrate host, or free in the environment, as these pathogens have a short season to develop into viable stages infectious to mammals, while obligate bacterial pathogens and especially viruses of mammals are sheltered from these extreme temperatures by the thermoregulation of their hosts.

### Freeze tolerance

For pathogens with a life stage outside the euthermic host during winter months, freeze tolerance is crucial to completing the life cycle of the organism. This characteristic is exemplified by *Trichinella *species. While the muscle larvae of *Trichinella spiralis*, the most common species of this roundworm threatening food supplies in temperate and tropical regions is highly susceptible to cold temperatures, *T. nativa*, the most prevalent species at high northern latitudes, has high freeze tolerance in carnivore hosts. Surviving freezing in the infected carcass allows transmission of larvae of *Trichinella nativa *during winter months. Apart from this general adaptation to freezing of muscle larvae, isolates from Alaska and Norway showed a clear correlation between low temperatures at the place of isolation with increased freeze tolerance [39].

Not only is the freeze tolerance higher at locations with colder mean January temperature as described above, but it also varies with host species. The muscle larvae lose their freeze tolerance in non-carnivore hosts such as mice. This is not a genomic change in the parasites, as the larvae of *T. nativ*a regain freeze tolerance when inoculated into foxes and other carnivores [40]. While rodents (the main reservoir in the environment) are often killed and eaten immediately, carnivores are more likely to freeze during winter, as they are not preyed upon with the same frequency as rodents. Therefore, the need for freeze tolerance in a rodent host is much lower than in a carnivore host. This example demonstrates that these pathogens can fine-tune their freeze tolerance to the host in which they encyst.

### Freeze avoidance

This strategy is employed by the winter tick *Dermacentor albipictus*, an ectoparasite of moose (*Alces alces*) and other ungulates. This tick is suspected to transmit anaplasmosis [41] and *Borrelia *species [42]. It survives the winter months on the host as nymphs and adults. The engorged females drop from the host in spring and lay eggs that develop into nymphs in the environment. In the fall, these ticks seek another warm blooded animal and spend the winter protected from the environment by host pelage and radiant body heat [43]. Attachment to the host is controlled by photoperiod, with 8 hours or less necessary for efficient attachment under laboratory conditions [44]. Winter tick range is controlled by summer temperatures of at least 865 Celsius degree days necessary for nymphal development and temperatures at the time engorged females drop from the infested hosts to the ground and lay their eggs [45]. Degree days are the accumulated product of time and temperature above a developmental threshold and therefore serve as a measure time above a minimum temperature necessary for development of many species in a pest control context. Photoperiod is known in other ticks to influence behavior of engorged females. Because the photoperiod in March at high latitudes is comparable to lower latitudes, female adults could drop onto snow-covered ground.

Currently, the winter tick has not been reported in the Arctic [43,45]. However, barren ground caribou can serve as experimental hosts and overlap habitat in some areas with moose that carry the tick. At this point it is not clear if a lack of contact or low temperatures have protected higher latitude caribou from this parasite. There is also a lack of more recent published studies that examine the winter tick with regards to a changing climate. Uncoupling of current temperature and photoperiod at key transition points during the year, could allow this tick, and similar pathogens to expand their range towards higher latitudes.

## Host-Pathogen-Environment Epidemiological Triad

To understand the potential for major changes in infectious disease dynamics due to climate change, one has to assess the interaction of pathogens and their host in the context of the environment. This Epidemiological Triad is a useful concept to better understand adaptation of host-pathogen interaction to the high latitude environment.

In the Arctic this triad is undergoing drastic cyclic changes on an annual basis, altering the complicated relationships throughout the year. The environment changes rapidly from a snow and ice covered insect free landscape to a lush green carpet with a high density of mosquito and other invertebrates present daylong. Concurrently, the Arctic ground squirrel changes its core body temperatures from below 0°C to 37°C, and *Francisella tularensis*, a major pathogen at high latitudes and the causative agent of tularemia, often shows high seasonality in outbreaks [46].

Living in the Arctic can be a strategy for mammals to avoid certain pathogens. One example for the absence of a specific ectoparasite on arctic mammals is the biting dog louse (*Trichodectes canis*) on wolves (*Canis lupus*). While this ectoparasite has been observed in the contiguous United States, it only recently has been detected in subarctic Alaska. It was first detected in south-central Alaska and has spread northwards just north of the Alaska Range moving further into interior Alaska. Currently wolves at higher latitudes are not infested, suggesting that environmental conditions prevent a further range expansion of this pathogen. Ecological parameters that correlated with infestation was a mean January temperature above -19°C [47].

The polar bear might be a similar example of pathogen avoidance by living in the high Arctic. Amstrup reports that in over 21 years of extensive work with polar bears he did not find evidence of ectoparasites [17]. Amstrup further reports only *Trichinella *as an endoparasite in wild polar bears. Rabies, a viral pathogen very common in the terrestrial arctic ecosystem has only been reported once in polar bears [48]. However, recent studies found significant levels of antibodies to canine distemper and other pathogens in polar bears [49-53]. Although antibodies to these pathogens have been documented in Alaska polar bears, the effects of such agents on the health of this species are still largely unknown, because baseline health data are limited, mostly due to the logistic constrains on access to studying the physiology of free ranging polar bears. As with other marine mammal species, carcasses of polar bears are not likely to be encountered since their habitat is remote and largely inaccessible to human surveillance.

Distemper viruses serve as an example of an arctic pathogen that causes outbreaks in species with a more southern habitat. Outbreaks of phocine distemper virus in European harbor seals in 1988 and 2002 were most likely caused by a virus transmitted from arctic phocid seal reservoir species to harbor seals in the North Sea with sympatric grey seals as potential links between the arctic reservoir and the host in the temperate climate zone [54,55].

The rabies virus is a pathogen that is well adapted to existence in this cold environment. The virus itself is tolerant of freezing and can maintain its viability for many years when in a frozen state. Arctic foxes (*Vulpes lagopus*) are principal hosts of this virus in the arctic and outbreaks occur every 3-4 years in northern and western Alaska. The ecology of the arctic fox in northern Alaska is quite different from foxes elsewhere, such as red (*Vulpes vulpes*) and gray (*Urocyon cinereoargenteus*) foxes at temperate latitudes of North America. In these southerly latitudes foxes maintain a defended territory throughout the year whereas arctic foxes in the North do so only during the breeding season. Following dispersal of young in late summer, territories break down and foxes roam in search of food [56,57]. Carcasses of marine and terrestrial mammals as well as landfills containing human garbage can be important food sources for winter survival of foxes. However, when foxes cease defending a territory infectious disease outbreaks are easily initiated because of the opportunity for close contact between animals at a food source. In addition, when foxes die of rabies in winter, frozen carcasses provide an excellent microenvironment for maintenance of the virus in tissues. Foxes and other scavengers attracted to these carcasses are then exposed to the virus through their buccal mucosa while gnawing on frozen flesh. Current work clearly illustrates the presence of rabies virus in numerous non-neural tissues harvested from clinically rabid arctic foxes (Gildehaus and Follmann, unpublished data).

However, rabies is neither endemic nor epizootic in interior Alaska despite the widespread presence of red foxes. Red foxes maintain more stable home ranges throughout the year in lower latitudes. Nothing is known about their ranges during winter in an Arctic environment. Maintaining movements within a restricted area compared to the widespread movements of arctic foxes in the North [56,57] greatly reduces encounters with other foxes thus, minimizing the potential for rabies transfer between conspecifics.

The long-term effect of climate change on rabies epizootiology in arctic Alaska can only be speculated upon. The freeze tolerance of the virus provides a source of infection when animals scavenge frozen carcasses. With climatic warming the seasonal duration of freezing temperatures in the Arctic will be shortened such that thawing carcasses will ultimately decompose to the extent that the virus will not be transmissible. The temperature at which this may occur is not known.

## Conclusion

A significant amount of research on seasonal changes in the environment and host has been conducted in the past and are ongoing. The knowledge concerning specific adaptations and mechanistic basis of seasonality in infectious diseases in the Arctic is more anecdotal rather than based on rigorous science in the field or laboratory. Additional studies are required in order to appreciate the potential of shifts in the interactions between the environment, pathogens and their host; especially when one considers the uncoupling of important environmental drivers. The anticipated changes, while unknown in extent or likelihood can potentially have drastic implications on mammals in the Arctic, including humans.

## Competing interests

The authors declare that they have no competing interests.

## Authors' contributions

TMO and KH conceived the overall idea of this review article. TMO, EHF and KH wrote the manuscript. All authors read and approved the manuscript.
